# Crabs Marine Waste—A Valuable Source of Chitosan: Tuning Chitosan Properties by Chitin Extraction Optimization

**DOI:** 10.3390/polym14214492

**Published:** 2022-10-24

**Authors:** Cristiana Luminița Gîjiu, Raluca Isopescu, Daniel Dinculescu, Maria Memecică, Manuela-Rossemary Apetroaei, Mirela Anton, Verginica Schröder, Ileana Rău

**Affiliations:** 1 Faculty of Chemical Engineering and Biotechnologies, University Politehnica of Bucharest, 011061 Bucharest, Romania; 2 Mircea cel Batran Naval Academy, Faculty of Navigation and Naval Management, 900218 Constanța, Romania; 3 Faculty of Pharmacy, Ovidius University of Constanta, 900470 Constanța, Romania

**Keywords:** chitin, chitosan, Taguchi method, chitin extraction optimization

## Abstract

Chitin extraction from crab shells was studied experimentally and optimized aiming to obtain chitosan with predefined deacetylation degree and molecular mass. To find out the optimum operating conditions that ensure the obtaining of a chitosan with highest deacetylation degree and specific molecular mass four parameters were varied: the concentration of NaOH and the temperature for deproteinization step, respectively HCl concentration and the number of acidic treatments for the demineralization stage. The experiment was carried on following Taguchi orthogonal array L9, and the best combination of factors was found using the desirability function approach. The optimization results showed that 5% NaOH concentration and low temperatures lead to a chitosan with high deacetylation degree. High molecular mass chitosan is obtained when a single step acidic treatment is used, while a chitosan with low molar mass is obtained for multiple acid contacts and higher HCl concentration.

## 1. Introduction

Over 70% of the total marine capture is used in the process industry, consequently it is remaining, as discarded material, significant quantities of crustacean and mollusk waste [1,2]. A large amount of crustacean waste is present on the Romanian Black Sea coast due to crustacean biomass released in natural, seasonal or after storm conditions in coastal areas. At the same time another important waste is represented by the shrimp waste, as a result of their consumption in the Romanian seaside restaurants. Over the time, many procedures for chitin extraction and chitosan preparation from marine waste were developed, most of them being used nowadays in the processing industry for chitin isolation and its derivatives obtaining [3]. Chitin could be isolated by biological extraction (fermentation), enzymatic hydrolysis [4,5] or by chemical procedures. The last procedure is often used in processing industry [6].

Biopolymers have a very high intrinsic molecular variability, due to their natural origin. The processes of extraction and treatment lead to different properties of samples from the same source [7]. The recommendations regarding the use of chitin and chitosan in different applications are also attended by the specifications of the sources, obtaining and processing, molecular properties and the biological activities induced [8,9,10,11,12].

A clear connection between the molecular morphology and molecular mass (MW) and interrelations with diversity of chitosan applications were highlighted [13,14]. Low MW of chitosan built 2D chains layouts, medium MW could be associated with single nanoparticles while the high MW chitosan generated nanoplates [7].

The mixtures between chitin/chitosan biopolymers [15] as well as with other polymers like collagen or gelatine [16,17] allow to obtain materials with new structural and mechanical properties for biomedical applications [15], or in other fields [12,18].

Data from the literature suggest that chitosan with low molar mass and pH < 6 is best suited to exert biological actions such as antimicrobial, antioxidant, antiviral activity, therapeutic and food protective properties [19,20,21]. An increased antimicrobial activity involves the formation of small intermolecular bonds associated with a higher degree of swelling [22]. The antibacterial activity of chitosan depends on its characteristics such as solubility in acidic or aqueous medium, but also on the particularities of the studied strains [23]. The antifungal activity correlates with the mechanism of action of chitosan through the positive amino groups that bind to the membrane phospholipids, negatively charged, from the structure of the cell walls of the fungi. Through this electrostatic interaction, a layer of polymer molecules is formed on the surface of the membranes that modifies the permeability of the membrane and limits the exchange of nutrients. It can also disrupt the structure of the membrane and by penetrating cells inhibits DNA/RNA nucleic acid activity or protein synthesis [24].

On the other side a high MW chitosan is recommended for drug delivery systems, tissue engineering or enzyme immobilizations [7].

In the same framework of the relationship between physical—chemical properties and biological effects it can be mentioned that the most common applications (wound healing [13], drug delivery [25], dietary ingredient [26,27], food preservative [28], wastewater treatment, molecule imprinting, metal reduction, textiles [29]) are associated with a chitosan with high deacetylation degree (DD). Chitosan with a DD around 85% or less than 80% is preferred for gene delivery or emulsifying agent or nanoparticle stabilization processes [7,14,30,31].

In their studies, Chuan D. et al. [14] showed the correlation between the changes of chitosan chemical properties with potential effects of biopolymer for gene delivery, co-vector, or a theragnostic agent, that can be used to diagnose and treat diseases simultaneously.

The aim of this paper is to present the optimization results concerning chitosan extraction from marine waste, particularly from crab waste. To the best of our knowledge, there are few studies in the literature on the optimization of chitosan extraction conditions from crab waste [32,33]. The research was conducted in order to highlight the importance of chitin extraction step on the final characteristics of chitosan, namely the specific molecular mass and the deacetylation degree.

## 2. Materials and Methods

### 2.1. Materials and Analytical Methods

The crustacean biomass subjected to the optimization process consisted of shells (exoskeletons) of two species of crabs, *Liocarcinus holsatus* (Fabricius, 1798) and *Pachygrapsus marmoratus* (J.C. Fabricius, 1787), which were harvested from the northern area of the Romanian seaside, namely the dam area, Năvodari during June–August 2021. The waste recovered from the natural environment was cleaned of sand, dust, organic material by successive washings with heated fresh water, dried in the oven at a temperature of 105 °C and turned into powder. The final powder, of a certain granulation, which was subject to recovery, contained 21% *L. holsatus* exoskeleton and 79% *P. marmoratus* shell.

The reagents used in the extraction procedure were hydrochloric acid (37%) p.a., purchased from Chemical Company Romania, and diluted to 4%, 6% and 8% according to the experimental design adopted, natrium hydroxide solutions (3%, 5%, 7%) prepared from NaOH pellets purchased from ChimReactiv S.R.L., with purity higher than 99.3%. The acetic acid used for chitosan solubilization, as well as ethanol and acetone (p.a.) were purchased from Sigma-Aldrich.

The deacetylation degree (DD) of chitosan was determined using the method described by Pădurețu et al. [33] namely potentiometric pH measurement, when the end of titration was indicated by the presence of two inflection points. The value of DD (%) was calculated according to [34] study (Equations (1) and (2)).

(1) $$DD\;(\%)=\frac{203\cdot Q}{1+42\cdot Q}$$

(2) $$Q=\frac{c_{M}\cdot \Delta V}{m}$$

where c_M_ represents the molar concentration of NaOH solution used for titration (mol/L); ΔV is the volume difference between the two inflection points (L); m is the mass of the analysed chitosan (g); 203 represents the molar mass of chitin (g/mol) and 42 is the molar mass of acetyl group (g/mol).

The molecular mass was estimated using the viscosimetric method according to the procedure described in [33].

Viscosimetry is the most utilized method to determine the molecular mass of chitosan due to its simplicity. This method is based on the relationship between the viscosimetric-average molecular mass, M_v_ (g/mol) and the intrinsic viscosity, [η] (mL/g) of the chitosan solution given by the well-known Mark–Houwink–Sakurada Equation (3), where K and α are constants which depend on the nature of the solvent, temperature, and chemical structure of the polymer.

(3) $$[\eta ]=K\cdot M_{v}^{\alpha }$$

Concentration, molar mass, solvent, temperature, shear rate, polymer chemical structure and the degree of chitosan deacetylation are all factors that influence the intrinsic viscosity of the polymer.

To determine the molecular mass of chitosan, a known mass was dissolved in a diluted acetic acid media and potassium chloride. The polymer was kept under stirring for 18 h to assure complete solubilization. The viscosimetric parameters were measured at room temperature using an Ubbelohde capillary viscometer.

The characterisation of the obtained chitosan was completed by infrared spectroscopy (Perkin Elmer Spectrum 100—ATR mode) and microscopic investigation. The extracted chitosan obtained by optimized chemical extraction has been identified by FTIR—ATR spectra and compared with two commercial chitosan samples from Sigma Aldrich: medium molecular mass (MMW—448877 product number) and low molecular mass (LMW—448869 product number). Chitosan samples subjected to IR analysis were brought to constant mass before analysis.

The fluorescent microscopy images were achieved using Optika B—350, blue filter (λ_ex_ = 450–490 nm; λ_em_ = 515–520 nm) and processed with Optikam Pro 3 Software.

In order to determine the fat binding capacity (FBC) a modified and improved method by Knorr and Rout was used (Equation (4)). Similarly, a modified and improved method of Wang and Knorr (Equation (5)) was used to calculate the water binding capacity (WBC). In both cases as equipment, a VELP—Digital IR Vortex mixer and a centrifuge were used. The solubility of chitosan samples was tested according to [33], by using a modified method of [35] (Equation (6)). The moisture and the ash content were determined following the methods described by [36]:

(4) $$FBC\;(\%)=\frac{m_{f,f}}{m_{i}}\times 100$$

where: m_f,f_ represents the final mass of chitosan sample after fat binding (g), and m_i_ is the initial mass of chitosan sample (g).

(5) $$WBC\;(\%)=\frac{m_{f,w}}{m_{i}}\times 100$$

where: m_f,w_ represents the final mass of chitosan sample after water binding (g), and m_i_ is the initial mass of chitosan sample (g).

(6) $$S\;(\%)=\frac{m_{i}-m_{f}}{m_{i}}\times 100$$

where: m_i_ is the initial mass of chitosan and m_f_ represents the final mass of chitosan, not dissolved.

### 2.2. Chitin/Chitosan Extraction Procedure

The industrial process of extracting chitosan from crustacean waste involves four classical stages: deproteinization, demineralization, discoloration, and deacetylation. In our previous studies [37], we showed that crab waste has a higher content of inorganic mineral salts than shrimps. Generally, the crustacean’s exoskeleton contains about 15–20% protein, 60–70% inorganic salts and 10–15% chitin, per dry mass.

**Figure 1:**
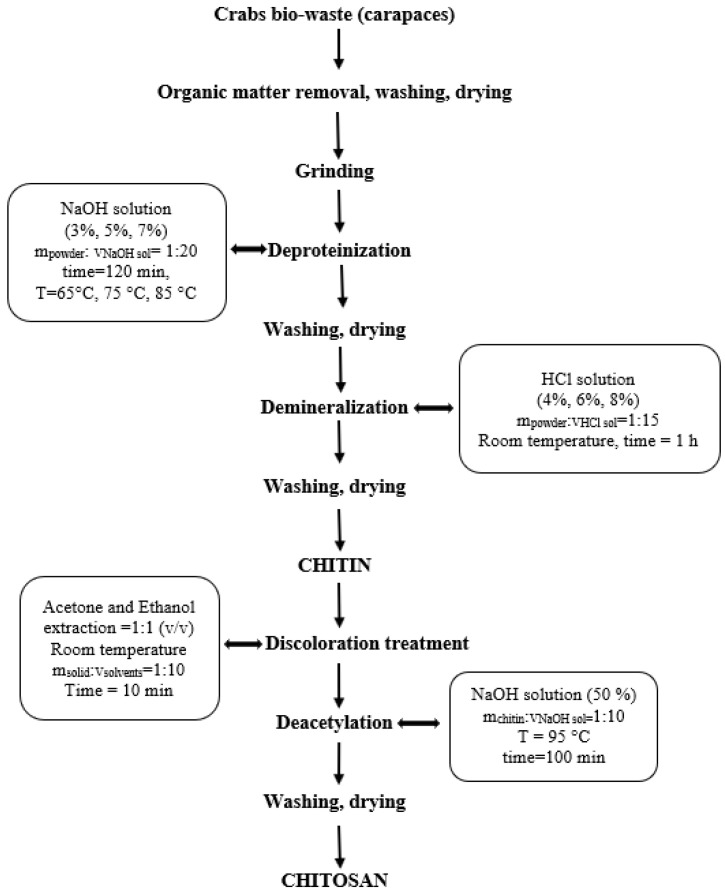
Chitosan extraction procedure.

The first stage of the procedure (Figure 1) is represented by the deproteinization of crab powder, which has the role of removing the protein content from the structure of the ground carcasses. This step consisted of immersion of a quantity of powder in an alkaline solution of NaOH, in constant ratio powder mass: NaOH solution volume = 1: 20 (m/v), working time of 120 min, and high working temperatures (65 ℃, 75 ℃, and 85 ℃), under constant stirring of 500 rpm.

The deproteinized powders were washed successively with distilled water until a neutral pH was reached, and the alkaline supernatants were removed. The deproteinized wet samples were dried overnight in the oven (T = 105 ± 2 °C) and weighed.

Demineralization is the second important step of the working procedure and consisted in immersing a quantity of deproteinated powder in HCl solution, in constant ratio powder mass: HCl solution volume = 1:15 (m/v), at room temperature, under constant stirring of 500 rpm.

After that the obtained samples were successively washed with distilled water and the acidic supernatants were successively removed, until a pH ≈ 6.5 was obtained in the last washing water. The chitinous samples were dried overnight in the oven at 105 ± 2 °C and weighed.

Since the crustacean exoskeleton naturally contains pigments such as astaxanthins and carotenoids, it was necessary to introduce a discoloration treatment to the chitinous samples obtained in the study. The discoloration procedure was performed at room temperature using a mixture of acetone and ethyl alcohol (1:1) in the ratio solid: solvent = 1:10 (m/v), and the material obtained was washed three times with distilled water for 10 min, to reach a neutral pH as mentioned above, dried in the oven (T = 105 ± 2 °C) and weighed.

In the extraction optimization study, the following factors were taken into consideration: the concentration of NaOH (%), the deproteinization temperature for deproteinization stage and the concentration of HCl (%) and the number of treatment repetitions with the HCl solution, for the demineralization process. These factors were considered very important and defining the chitin extraction stage.

The deacetylation procedure followed is described in our previous paper [33], related to chitosan extraction from crab waste where the optimal recommended deacetylation conditions to obtain chitosan with a relatively high degree of deacetylation were determined. Therefore, the chitin obtained was soaked in a 50% NaOH concentration solution, in a settled solid -to- solvent ratio (*w*/*v*) of 1: 10, for 100 min, at temperature of 95 °C.

### 2.3. Optimization of Chitin Extraction from Crab’s Shell

The optimization of chitin extraction from marine wastes was extensively studied using various experimental and numerical methods. The chemical process of extraction is mainly studied using HCl for demineralization and NaOH for deproteinization, at various concentrations and temperatures. The best operating conditions, based on 27 experimental runs, for chitin extraction from shrimps, in terms of yield and FT-NIR spectrum, reported in [38] correspond to 3% HCl at 25 °C for 1 h and 50% NaOH at 110 °C and 3 h. Statistical methods for process optimization implied the experimental design techniques. Box-Behnken design was used to optimize the enzymatic extraction of chitin from shrimp shell, namely the deproteinization degree, varying the incubation time, temperature and enzyme/substrate ratio [39]. Chitin extraction from shrimp shells by biological treatment was also optimized by [40] aiming to improve the yield of extraction. A Plackett—Burman design was used to screen the influence of eight factors, and subsequently a central composite design focused on the most important 4 factors established the best operating conditions.

The chitin extraction steps are also responsible for the final chitosan properties [41] and the deterioration of chitin molecules during the alkaline and acid treatments may lead to a chitosan of poor quality [42]. In the present study, the extraction of chitin from crab shells was optimized in terms of optimal final chitosan characteristics (deacetylation degree and molecular mass) using an orthogonal experimental program according to the Taguchi method.

The orthogonal experimental matrices defined by Taguchi allow the exploration of many parameters, at two or more levels, in a limited number of experiments. The statistical analysis of these data is mainly based on the investigation of the mean value of the output for each level of a given factor, regardless of the values of other factors. Due to this feature, the Taguchi method is often used in the analysis and optimization of processes where the number of factors that influence the results is high, and the possibility of carrying out practical experiments is limited. Taguchi optimization method was successfully applied to find favorable operating conditions for complex extraction processes including chitin extraction from marine wastes [43,44]. Taguchi method is formulated for the optimization of a single objective function and therefore the optimization of multiple quality characteristics requires a combination of techniques. In the present study, where the optimization goal was to find out the best chitin extraction conditions that would lead to a chitosan with high degree of deacetylation and predefined molecular mass, the desirability function approach was chosen. The key step in this hybrid method is to transform each objective function into a unitless desirability function di which may vary between 0 (the worst case) and 1 (the best situation) and to lump these individual functions into a global desirability D calculated as geometric mean (relation 7):

(7) $$D={\left(d_{1}^{n_{1}}\cdot d_{2}^{n_{2}}\ldots \cdot d_{k}^{n_{k}}\right)}^{{}^{\frac{1}{\sum_{j=1}^{k}n_{j}}}}$$

The powers n_j_ stand for the relative importance in the lumped function of each individual desirability, calculated with:

(8) $$d_{i}=\left\{\begin{array}{cc} 0 & y_{i}\leq L\\ \left(\frac{y_{i}-L_{i}}{H_{i}-L_{i}}\right) & L<y_{i}<H\\ 1 & y_{i}>H \end{array}\right\}\;\mathrm{for}\;\mathrm{maximization}\;$$

(9) $$d_{i}=\left\{\begin{array}{cc} 1 & y_{i}\leq L\\ \left(\frac{H-y_{i}}{H-L}\right) & L<y_{i}<H\\ 0 & y_{i}>H \end{array}\right\}\;\mathrm{for}\;\mathrm{minimization}\;$$

where L is the lowest acceptable value, H the highest one, and y_i_ is the current response in the data set.

## 3. Results

### 3.1. Optimization Chitin/Chitosan Extraction

The factors considered to influence the extraction are the HCl concentration and number of acidic treatments in the demineralization step and NaOH concentration and temperature for the deproteinization stage. The four factors were investigated at three levels as mentioned in Table 1.

The experiments were performed according to the L9 orthogonal array and are presented in Table 2.

**Figure 2:**
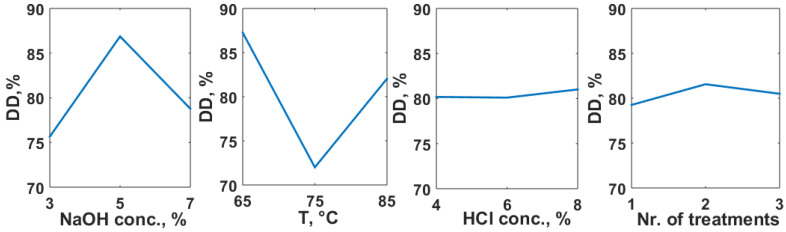
Influence of the operating conditions in chitin extraction upon the deacetylation degree of chitosan.

The first attempt was performed to find out the optimum chitin extraction conditions to finally obtain a chitosan with the highest deacetylation degree, as required in all practical applications. Applying the Taguchi method, the influence of each factor has been put in evidence (Figure 2).

As it can be noticed, the variation of the first two factors referring to the deproteinization stage is more important than the modification of the demineralization conditions. This fact is sustained also by the variance analysis (ANOVA), from which resulted a contribution of NaOH concentration of 35.19%, and of temperature of 63.15%, while the contributions of HCl concentration and the number of acidic treatments were only 0.27% and 1.40%, respectively.

The optimum operating conditions correspond to: NaOH solution concentration: 5%, alkaline treatment temperature: 65 °C, HCl solution concentration: 8% and 2 acidic treatments. As the cumulate contribution of factors F3 and F4 is below 2%, the control of chitin extraction may be ensured by the operating conditions in the deproteinization step.

The estimation of expected deacetylation degree in the optimum operating conditions, estimated by the Taguchi method, may be calculated with the relation (10):

(10) $$DD\;\%={\bar{F}}_{1}+{\bar{F}}_{2}+{\bar{F}}_{3}+{\bar{F}}_{4}-3\cdot \bar{T}=94.5$$

where

${\bar{F}}_{1}$ is the mean deacetylation degree for factor F1 (NaOH concentration) at level 2

${\bar{F}}_{2}$ is the mean deacetylation degree for factor F2 (temperature) at level 1

${\bar{F}}_{3}$ is the mean deacetylation degree for factor F3 (HCl concentration) at level 3

${\bar{F}}_{4}$ is the mean deacetylation degree for factor F4 (number of acidic treatments) at level 2

$\bar{T}$ is the mean of deacetylation degree over all experiments

The estimated optimal value for DD is 94.5%, slightly better than the best value obtained experimentally (run 4 in Table 2) where NaOH concentration and temperature correspond to the optimum conditions, and only the operating conditions for acid treatment are different. As the build-up of optimum conditions when a high DD is expected is less than 4% influenced by the demineralisation step, 5% NaOH solutions and a temperature of 75 °C in the deproteinization step may be recommended to attain the goal. When both deacetylation degree and molecular mass of chitosan must be as high as possible, the desirability function was used when applying the Taguchi method. The two criteria were considered to be equally important and the powers n_j_ in relation (7) were set n_1_ = n_2_ = 1.

The individual desirability and the final desirability function for the maximization of the deacetylation degree and molar mass are presented in Table 3.

**Figure 3:**
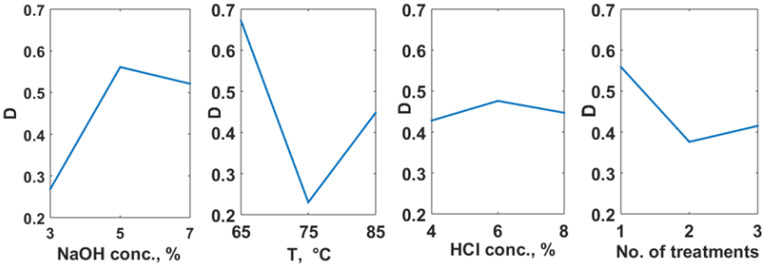
Factors influence for chitin extraction aiming to obtain maximum deacetylation degree and maximum molar mass in chitosan.

As it is shown in Figure 3, the concentration of the NaOH solution and temperature are the factors with the highest contribution (30.02%, and 58.15%, respectively), but the number of acidic treatments has also a significant contribution (11.13%), while HCl concentration has a contribution of only 0.70%, as revealed by ANOVA. The optimal working conditions correspond to a NaOH concentration of 5%, a temperature of 65 °C, a HCl concentration of 6% and a minimum number of repetitions (one treatment). This is in good agreement with the assumption that a single acidic treatment would prevent the rupture of chitin molecules.

A validation experiment was performed for the optimum operating conditions identified (NaOH concentration 5%, temperature 65 °C—for deproteinization step, HCl concentration 6%, and 1 acidic treatment—for demineralization stage). The desirability function calculated for the deacetylation degree (91.95%) and chitosan molar mass (535.39 kDa) in the validation experiment is 0.97, greater than the values in Table 3.

The extraction conditions to obtain a chitosan with high degree of deacetylation but with low molar mass were also investigated using the method of the desirability function. The individual desirability and the global desirability, D, calculated for these two objectives are presented in Table 4.

**Figure 4:**
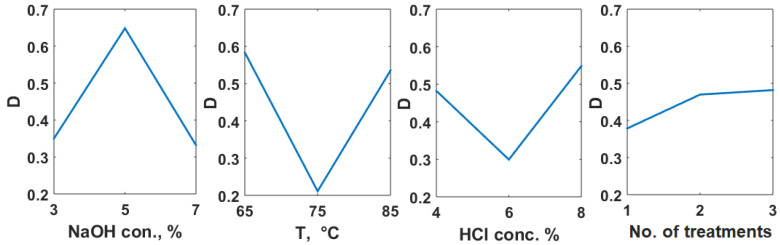
Factors influence for chitin extraction for maximum deacetylation degree and minimum molar mass in chitosan.

The study of the influence of the investigated factors (Figure 4) shows a more important contribution of the acidic treatment (8.845%). The contributions of the other factors were 36.03% (NaOH concentration), 29.79% (temperature), and 25.37% (number of acidic treatments). The optimum HCl concentration is 8% and the number of treatments is 3. A more aggressive acidic treatment enables to obtain a chitosan with lower molecular mass.

As contribution values show, the contribution of the demineralization step is comparable with the influence of the deproteinization step if a chitosan with high DD and low molecular mas is desired.

### 3.2. Chitosan Caracterization

The data obtained on the properties of chitosan are presented in Table 5. At the first sight it can be affirmed that the obtained chitosan has a good solubility and a low content in inorganic materials (low ash). As expected, the extraction conditions have consequences on the chitosan characteristics. However, all obtained chitosan presents good fat binding properties and a relatively low moisture despite the high-water binding capacity.

The characterization of chitosan samples was carried out spectral, by microscopy in epifluorescence and analytically. The data obtained by epifluorescence microscopy showed that the method of obtaining chitosan has an influence on it.

**Figure 5:**
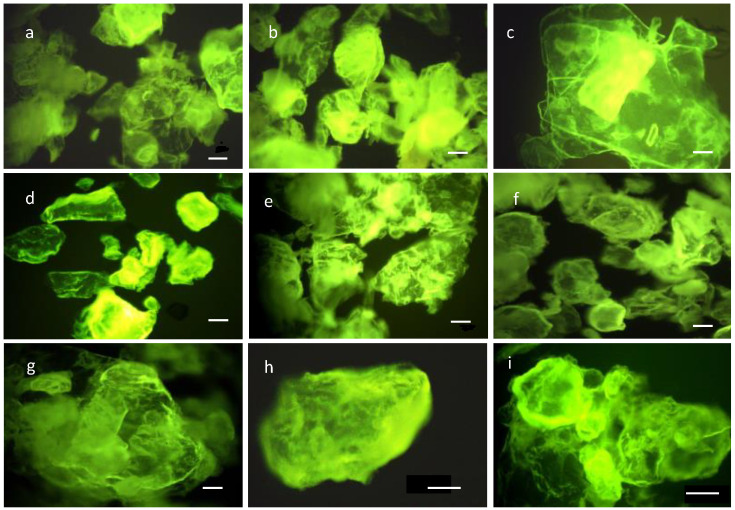
Epifluorescence images of chitosan powder prepared from 1–9 parameters (Table 5) (**a**–**g**) ×100; (**h**,**i**) ×200 magnification, scale bar = 100 μm.

The chitosan particles morphology indicated a variety of types (Figure 5a–i). The particles characterization showed from images two detailed shapes: glomerular and lamellar with variable size between 100–1200 µm. The image of the sample 2 analysis presented irregular, aggregated and amorphous shapes of the particles (Figure 5b). The particle from this sample included the elements of different consistency and colours, in the 30–40% proportions, appearance due to impurities. The gathered morphotypes in the large formations, compact and with rounded edges were observed in the 6th (Figure 5h) and 7th samples (Figure 5g). These particles were without impurities.

At the same time the large flakes shapes were found in the samples 3 and 9 (Figure 5c,i), obtained using higher temperatures (85 °C) conditions while the globular structures with rounded edges it’s possible to be associated with the lower temperature (65–75 °C) projected in the experiment.

The image analysis and the chitosan characterization properties indicated the relationship between physical-chemical conditions (pH, temperature °C, DD, MW), and the ability to form spherical or flakes particles. For instance, the chitosan with high DD presents smaller size of microparticles and lower aggregation effect of chitosan powder. Chitosan with a higher molecular weight favours the formation of large particles in a flake like shape.

**Figure 6:**
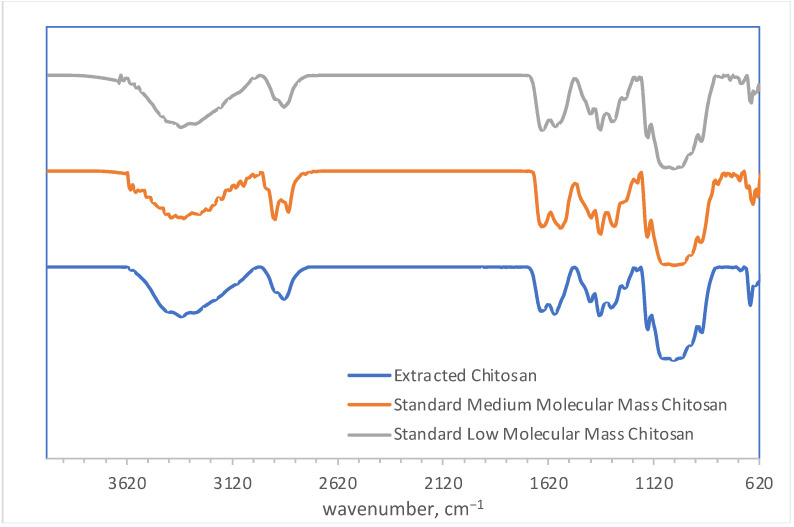
Infrared spectrum of extracted chitosan compared to infrared spectra of medium and low molecular mass chitosan.

As it can be seen in Figure 6 a broad absorption band appeared at about 3420–3220 cm**^−^**^1^ was registered in all three samples. This band was assigned to O-H vibrations of CH_2_OH or O-H groups from glucoside ring implicated in intramolecular hydrogen bonds [45]. These vibrations could overlap with the N-H stretching vibrations of primary amines [46].

By comparing the three spectra obtained, the characteristic absorption peaks registered at 1645 cm**^−^**^1^ (extracted chitosan), 1643 cm**^−^**^1^ (low molecular mass chitosan—LMW) and 1651 cm**^−^**^1^ (medium molecular mass chitosan—MMW) were attributed to the stretching of C=O in amide bond (Amide I) and CO-NH bending vibration respectively. The chitosan characteristic peaks corresponding to NH_2_ bending in NHCOCH_3_ group (Amide II band) [47] were recorded at 1585 cm**^−^**^1^ (extracted chitosan), 1581 cm**^−^**^1^ (LMW) and 1559 cm**^−^**^1^ (MMW) respectively. The peaks at 1417 cm**^−^**^1^ (extracted chitosan), 1416 cm**^−^**^1^ (LMW) and 1414 cm**^−^**^1^ (MMW) were assigned to OH bending vibrations in CH_2_OH group [36].

## 4. Conclusions

The extraction procedure conditions were proved to have importance on the chitin/chitosan properties, i.e., the chitosan powder particle shape, sizes and aggregation being induced by the temperature in the deproteinization process.

At the same time our study revealed, on one side, the importance of deproteinization treatment upon the degree of deacetylation. For 5% NaOH concentration and low temperatures a maximum degree of deacetylation may be reached, regardless of the conditions in the demineralization step.

When the molar mass of chitosan is an additional criterion, the acid treatment becomes important. A single-step acidic treatment is recommended for obtaining a high molar mass, while multiple acid contacts and higher HCl concentration are required to obtain a chitosan with low molar mass.

## Figures and Tables

**Table 1:** Factor levels.

Factor	Levels		
	Level 1	Level 2	Level 3
NaOH concentration (%)	3	5	7
Temperature, °C	65	75	85
HCl concentration (%)	4	6	8
Number of acidic treatments	1	2	3

**Table 2:** Experimental plan and obtained chitosan characteristics.

Run No.	Deproteinization		Demineralization		Characteristics of Obtained Chitosan	
	NaOH Conc., %	Temperature, °C	HCl Conc., %	No of Acid Treatments	Deacetylation Degree (DD), %	Molar Mass, kDa
1	3	65	4	1	81	331
2	3	75	6	2	68	111
3	3	85	8	3	78	156
4	5	65	6	3	93	314
5	5	75	8	1	78	287
6	5	85	4	2	89	198
7	7	65	8	2	87	309
8	7	75	4	3	70	350
9	7	85	6	1	79	450

**Table 3:** Individual and global desirability for maximum deacetylation degree and maximum molar mass.

Run No.	NaOH Conc. %	T, °C	HCl Conc. %	No of Acid Treatments	Y1 Molar Mass kDa	Y2 (DD) %	d_1_	d_2_	D
1	3	65	4	1	331	81	0.649	0.513	0.577
2	3	75	6	2	111	68	0.000	0.000	0.000
3	3	85	8	3	156	78	0.133	0.389	0.228
4	5	65	6	3	314	93	0.598	1.000	0.773
5	5	75	8	1	287	78	0.517	0.387	0.447
6	5	85	4	2	198	89	0.255	0.839	0.462
7	7	65	8	2	309	87	0.583	0.759	0.665
8	7	75	4	3	350	70	0.704	0.084	0.244
9	7	85	6	1	450	79	1.000	0.427	0.654

**Table 4:** Individual and global desirability for maximum deacetylation degree and minimum molar mass.

Run No.	NaOH Conc. %	T, °C	HCl Conc., %	No of Acid Treatments	Y1 Molar Mass kDa	Y2 DD %	d_1_	d_2_	D
1	3	65	4	1	331	81	0.351	0.513	0.424
2	3	75	6	2	111	68	1.000	0.000	0.000
3	3	85	8	3	156	78	0.867	0.389	0.581
4	5	65	6	3	314	93	0.402	1.000	0.634
5	5	75	8	1	287	78	0.483	0.387	0.432
6	5	85	4	2	198	89	0.745	0.839	0.791
7	7	65	8	2	309	87	0.417	0.759	0.563
8	7	75	4	3	350	70	0.296	0.084	0.158
9	7	85	6	1	450	79	0.000	0.427	0.000

**Table 5:** Chitosan full characterisation.

Sample	Characterisation						
	Deacetylation Degree, %	Molecular Mass, kDa	Moisture, %	Fat binding Capacity, %	Water Binding Capacity, %	Solubility, %	Ash, %
1	81	331	8.81	673.88	993.11	98.49	3.01
2	68	111	6.86	572.03	618.74	95.33	16.97
3	78	156	9.31	624.20	821.86	99.51	4.04
4	93	314	8.44	688.31	706.18	96.09	2.59
5	78	287	9.04	501.35	698.81	97.02	6.34
6	89	198	8.67	661.32	890.84	98.53	6.45
7	87	309	10.19	809.09	892.66	92.45	4.33
8	70	350	9.30	661.50	848.83	97.02	4.27
9	79	450	9.15	743.47	882.31	90.23	8.44
**Validation Experiment**	92	535	4.53	532.69	890.84	93.60	3.90

## Data Availability

Not applicable.
